# Isolation of Carbapenem and Colistin Resistant Gram-Negative Bacteria Colonizing Immunocompromised SARS-CoV-2 Patients Admitted to Some Libyan Hospitals

**DOI:** 10.1128/spectrum.02972-22

**Published:** 2023-04-12

**Authors:** Khouloud Slimene, Asrra A. Ali, Elhussan A. Mohamed, Allaaeddin El Salabi, Faraj S. Suliman, Agela A. Elbadri, Fadi F. El-fertas, Ahmed El-awjly, Salah A. Shokri, Jean-Marc Rolain, Chedly Chouchani

**Affiliations:** a Université Aix-Marseille, IRD, APHM, MEPHI, Faculté de Médecine et de Pharmacie, Marseille Cedex 05, France; b IHU Méditerranée Infection, Marseille Cedex 05, France; c Laboratoire des Microorganismes et Biomolécules Actives, Faculté des Sciences de Tunis, Campus Universitaire, 2098 El-Manar II, Université de Tunis El-Manar, Tunis, Tunisie; d Laboratoire de Recherche des Sciences et Technologies de l’Environnement, Institut Supérieur des Sciences et Technologies de l’Environnement de Borj-Cedria, BP-1003, Hammam-Lif 2050, Université de Carthage, Tunisie; e Unité de Service en Commun pour la Recherche Plateforme Génomique Institut Supérieur des Sciences et Technologies de l’Environnement de Borj-Cedria, BP-1003, Hammam-Lif 2050, Université de Carthage, Tunisie; f Department of Environmental Health, Faculty of Public Health, University of Benghazi, Benghazi, Libya; g Department of Community Medicine, Omar Al-Mukhtar University, El-Beyda, Libya; h Almansoura Isolation Department of COVID-19 Patients, Shahat Teaching Hospital for Chest Diseases and Tuberculosis, Shahat, Libya; i Department of Medicine, Faculty of Medicine, University of Benghazi, Benghazi, Libya; j Alhawari Hospital, Benghazi, Libya; k Sterilization Service Department, Benghazi Medical Center, Benghazi, Libya; l Department of Microbiology, Faculty of Science, Misurata University, Misurata, Libya; Vanderbilt University Medical Center

**Keywords:** SARS-CoV-2, colonization, bacteria, carbapenemase, colistin resistance, Libya, COVID-19, infection

## Abstract

The emergence of severe acute respiratory syndrome coronavirus 2 (SARS-CoV-2) has had a devastating effect, globally. We describe, for the first time, the occurrence of carbapenem-resistant bacteria colonizing SARS-CoV-2 patients who developed hospital-associated infections with carbapenemase-producing, Gram-negative bacteria at some isolation centers of SARS-CoV-2 in the eastern part of Libya. In total, at first, 109 samples were collected from 43 patients, with the samples being recovered from oral (*n* = 35), nasal (*n* = 45), and rectal (*n* = 29) cavities. Strain identification was performed via matrix assisted laser desorption ionization-time of flight (MALDI-TOF). Antibiotic susceptibility testing was carried out on Mueller-Hinton agar, using the standard disk diffusion method. MIC determination was confirmed via E-TEST and microdilution standard methods. A molecular study was carried out to characterize the carbapenem and colistin resistance in Gram-negative bacterial strains. All of the positive results were confirmed via sequencing. Klebsiella pneumoniae (*n* = 32), Citrobacter freundii (*n* = 21), Escherichia coli (*n* = 7), and Acinetobacter baumannii (*n* = 21) were the predominant isolated bacteria. Gram-negative isolates were multidrug-resistant and carried different carbapenem resistance-associated genes, including NDM-1 (56/119; 47.05%), OXA-48 (15/119; 12.60%), OXA-23 (19/119; 15.96%), VIM (10/119; 8.40%), and the colistin resistance mobile gene mcr-1 (4/119; 3.36%). The overuse of antimicrobials, particularly carbapenem antibiotics, during the SARS-CoV-2 pandemic has led to the emergence of multidrug-resistant bacteria, mainly K. pneumoniae, A. baumannii, and colistin-resistant E. coli strains. Increased surveillance as well as the rational use of carbapenem antibiotics and, recently, colistin are required to reduce the propagation of multidrug-resistant strains and to optimally maintain the efficacy of these antibiotics.

**IMPORTANCE** In this work, we describe, for the first time, the occurrence of carbapenem-resistant bacteria colonizing COVID-19 patients who developed hospital-associated infections with carbapenemase-producing, Gram-negative bacteria at some isolation centers of COVID-19 in the eastern part of Libya. Our results confirmed that the overuse of antimicrobials, such as carbapenem antibiotics, during the COVID-19 pandemic has led to the emergence of multidrug-resistant bacteria, mainly K. pneumoniae and A. baumannii, as well as colistin resistance.

## INTRODUCTION

In December of 2019, a new virus called SARS-CoV-2 emerged in the world and caused a high risk to health care settings, globally. This consequently led to an increase in infections and mortality levels. The World Health Organization (WHO) announced a global emergency on January 30, 2020, concerning the outbreak of a novel coronavirus in Wuhan, a city located in China’s Hubei province (1). Later, on February 24, 2020, the WHO agreed that SARS-CoV-2 has the potential to spread on a global scale and to lead to a pandemic outbreak (1). Thereafter, on March 11, 2020, the WHO announced that SARS-CoV-2 was a pandemic (1).

Previous studies have described that the main strategies proposed by the WHO to reduce SARS-CoV-2 are the uses of hand sanitizers and disinfectants, as much as possible (2). These products are rich in alcohol, quaternary ammonium compounds, phenols, hydrogen peroxide, and surfactants at various frequencies, concentrations, and doses, which can damage microbial DNA (3). Consequently, it can be deduced that the misuse of antimicrobial agents may contribute to the occurrence and spread of antimicrobial resistance (AMR) via mutagenic mechanisms that induce the instability of the microbial genome. AMR is a serious and urgent public health problem. It has been estimated that by 2050, AMR will be the cause of death for 10 million people and, in addition, will cost up to $100 trillion (4).

The spread of SARS-CoV-2 around the world has been followed by an increase of antibiotic consumption. In fact, antibiotics were administered in almost 70% of SARS-CoV-2-related hospital admissions and in 80 to 100% of SARS-CoV-2-related ICU admissions (5). The rising use of antibiotics is the leading cause of the emergence and spread of AMR, which is a global health challenge (6).

One report from Paris, France, suggests that between mid-March and mid-May of 2021, as well as during the third SARS-CoV-2 pandemic wave in Paris, an outbreak of Klebsiella pneumoniae producing the carbapenemase NDM-1 (NDM-1 K. pneumoniae) occurred in the intensive care unit (ICU) (7). A meta-analysis that was conducted by Langford and coauthors and published in 2020 reported that out of 3,338 patients who were hospitalized with SARS-CoV-2, a secondary bacterial infection was found in 6.9% of the cases and was more prevalent in critically ill patients (13.8%) (5). The most prevalent bacteria were Mycoplasma pneumoniae and Gram-negative pathogens, such as extended spectrum beta-lactamase (ESBL)-producing Klebsiella pneumoniae, ESBL-positive Pseudomonas aeruginosa, carbapenem-resistant Klebsiella pneumoniae, and carbapenem-resistant Acinetobacter baumannii (8).

Here, we describe, for the first time, the occurrence of carbapenem-resistant bacteria colonizing SARS-CoV-2 patients who developed hospital-associated infections caused by carbapenemase-producing, Gram-negative bacteria at some isolation centers of SARS-CoV-2 in the eastern part of Libya.

## RESULTS

### Bacterial isolates.

Samples were collected from SARS-CoV-2 patients during the third wave of SARS-CoV-2 from May to June of 2021 in Libya. Among the 109 swabs taken, only 70 swabs yielded bacterial isolates when grown on culture media (MacConkey agar with ertapenem) (64.22%), and Gram-negative bacterial strains accounted for 119 of the obtained isolates. For MacConkey agar with cefotaxime, 86 samples were grown, and a total of 144 pure strains were obtained. On LBJMR media, only 12 isolates were obtained (strains with intrinsic resistance were eliminated from this study). The strains were from immunocompromised patients with chronic diseases (diabetes mellitus, hypertension, and cancer) (data not shown).

**FIG 1:**
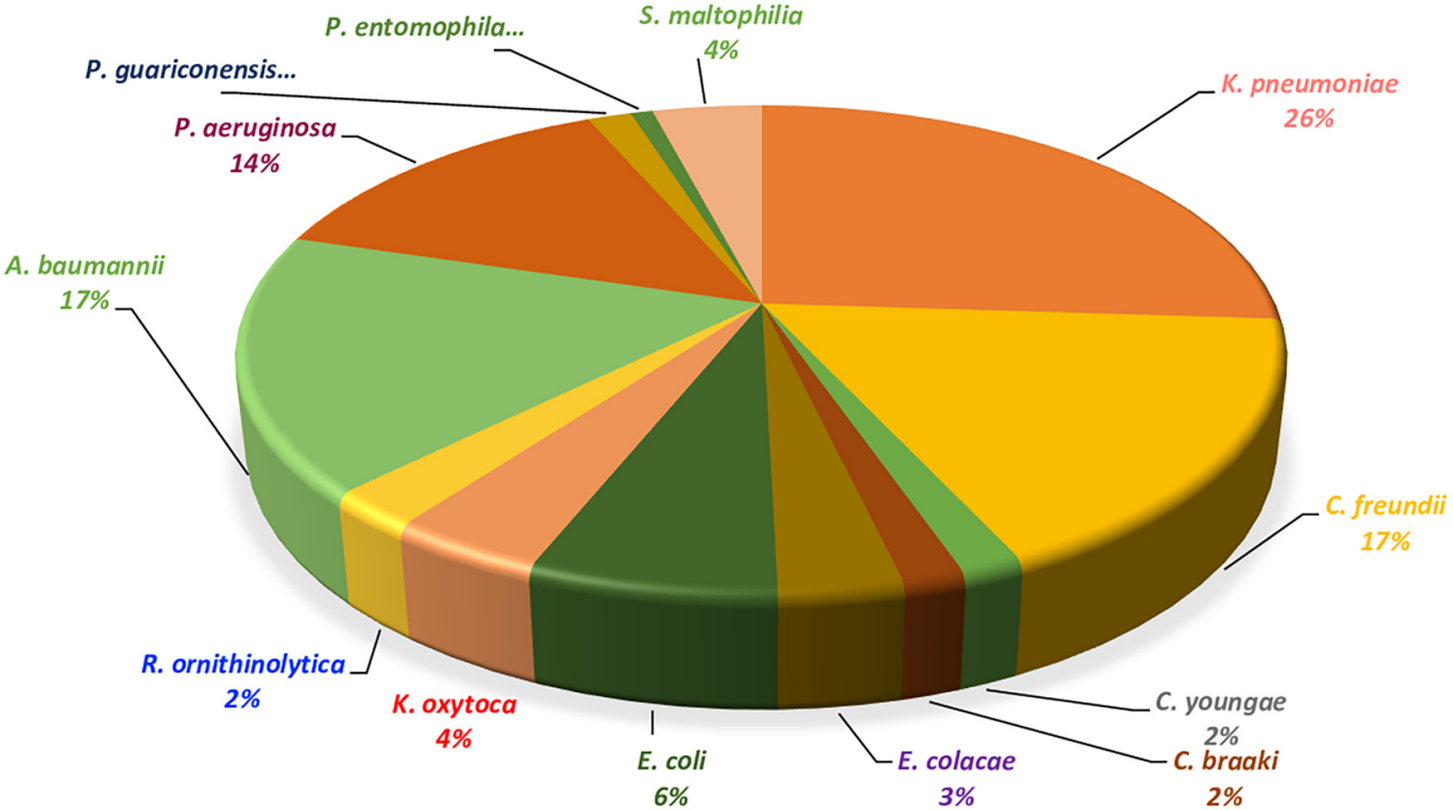
Gram-negative bacteria strains distribution.

In the current study, we focused on the strains identified on MacConkey agar with ertapenem and LBJMR media. Identification via MALDI-TOF showed that these strains were as follows: *Enterobacterales* (*n* = 79): (*K. pneumoniae* [*n* = 32], C. freundii [*n* = 21], E. coli [*n* = 8], K. oxytoca [*n* = 5], *R. ornithinolytica* [*n* = 3], Enterobacter cloacae [*n* = 4], *C. youngae* [*n* = 2], and *C. braakii* [*n* = 2]). The non-*Enterobacterales* (*n* = 46) are distributed as below: A. baumannii (*n* = 21), P. aeruginosa (*n* = 17), *Stentrophomonas maltophilia* (*n* = 5), *P. guariconensis* (*n* = 2), and *P. entomophila* (*n* = 1). The distribution of the strains is illustrated in Fig. 1, and the clinical and isolate information are summarized (data not shown).

### Phenotypic characterization of antibiotic resistance.

**FIG 2:**
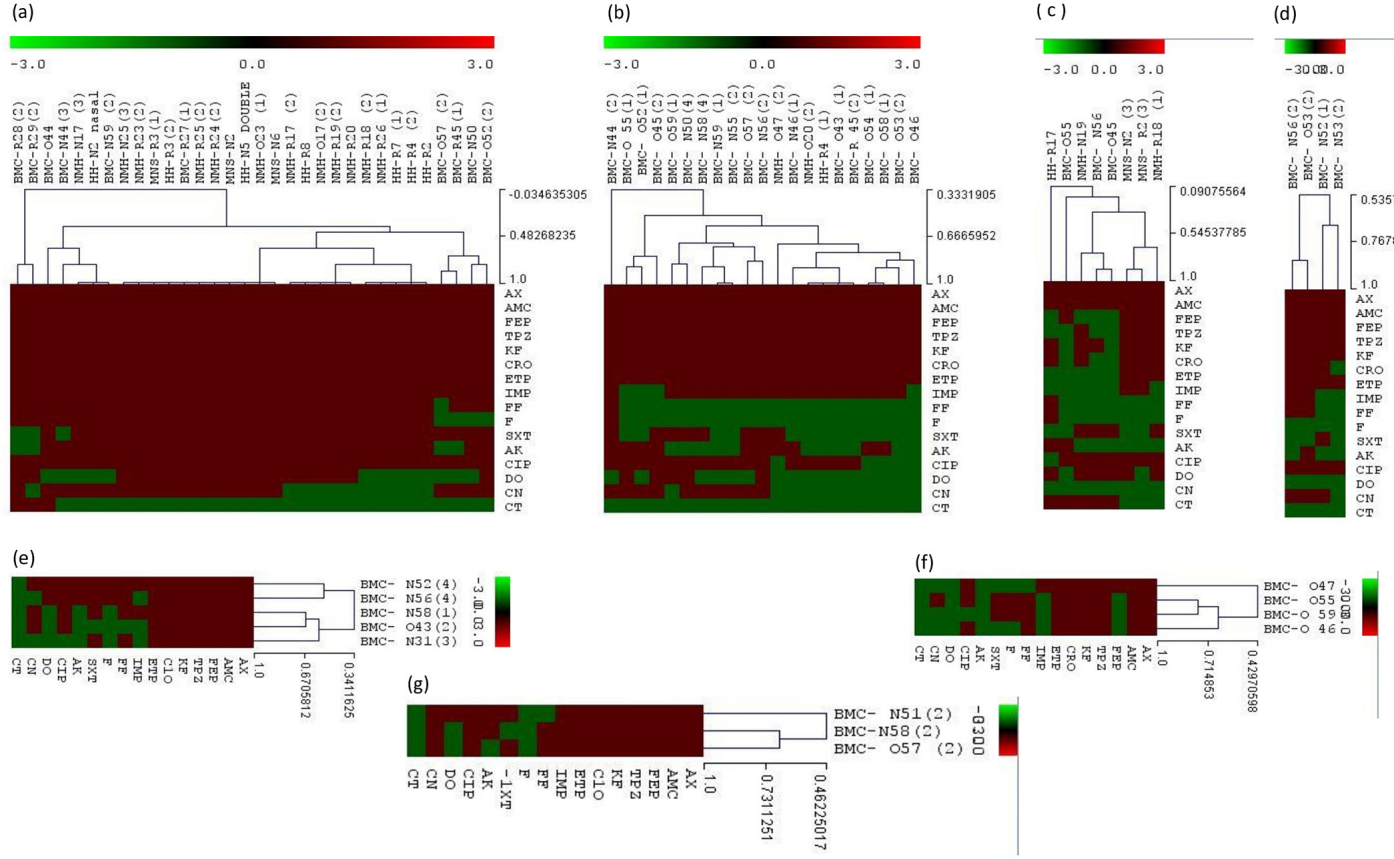
Hierarchical clustering of antibiotic susceptibility profiles (disk diffusion method), representing the activity of the 16 antibiotics tested against the 79 *Enterobacterales* strains isolated in this study. (A): K. pneumoniae, (B): C. freundii, (C): E. coli, (D): *C. youngae*; *C. braakii*, (E): K. oxytoca, (F): E. cloacae, (G): *R. ornithinolytica*. AX: amoxicillin, AMC: amoxicillin + clavulanic acid, FEP: cefepime, TPZ: piperacillin + tazobactam, KF: cefalotin, CRO: ceftriaxone, ETP: ertapenem, IMP: imipenem; FF: fosfomycin, F: nitrofurantoin, SXT: trimethoprim/sulfamethoxazole, AK: amikacin, CIP: ciprofloxacin, DO: doxycyclin, CT: colistin, CN: gentamicin.

Antibiotic susceptibility testing for *Enterobacterales* reported a high resistance to the β-lactam family (100%) (Fig. 2). K. pneumoniae was the most prevalent species, and it was followed by C. freundii, *R. ornithinolytica*, K. oxytoca, *C. braakii*, *C. youngae*, E. cloacae, and E. coli. K. pneumoniae were resistant to aminoglycosides, with 50% and 87.5% for gentamicin and amikacin, respectively. Resistance to quinolones, sulfonamides, nitrofurans, and cyclines was observed in 100%, 90.62%, 87.5%, and 31.25% of the strains, respectively. However, colistin was the most active antibiotic on most tested strains (12.5%).

For C. freundii, resistance to gentamicin and amikacin was reported to be 47.6% and 42.85%, respectively, whereas resistance to quinolones and sulfonamides was reported to be 66.66% and 38.09% of the strains. However, fosfomycin and nitrofurans were the most active antibiotics on the tested strains (4.76%). For other species, resistance was mainly assigned to quinolones, aminoglycosides, sulfamides, nitrofurans, and fosfomycin. Doxycyclin and colistin seem to be the most effective molecules against these pathogens.

The imipenem E-TEST showed that all K. pneumoniae were resistant to imipenem, with MICs ranging from 3 mg/L to >32 mg/L, and ertapenem, with MICs ranging from 1.5 mg/L to >32 mg/L. 13 strains had a MIC of >32 mg/L for imipenem, and 24 strains had a MIC of >32 mg/L for ertapenem. Concerning C. freundii, the imipenem E-TEST revealed that strains were highly resistant to ertapenem, with MICs ranging from 1 mg/L to >32 mg/L, and to imipenem, with MICs ranging from 2 mg/L to >32 mg/L.

**FIG 3:**
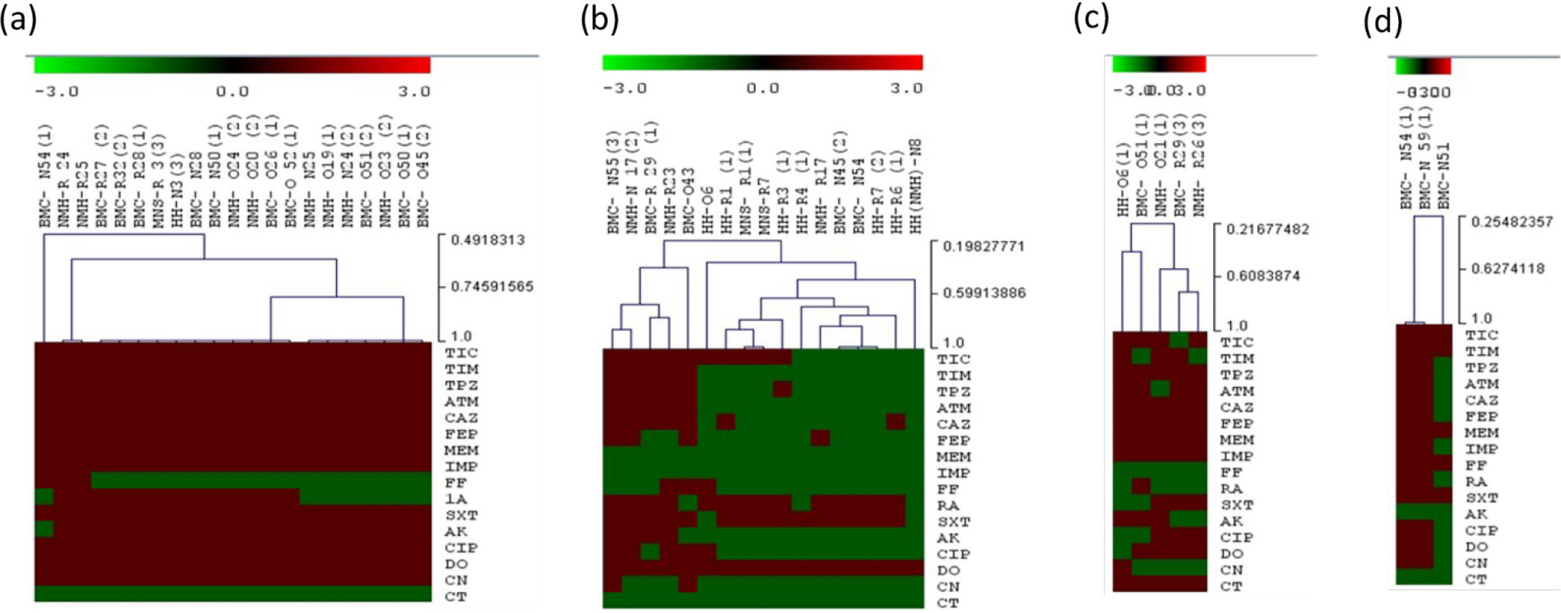
Hierarchical clustering of antibiotic susceptibility profiles (disk diffusion method) representing the activity of the 16 antibiotics tested against 42 *non-Enterobacteriaceae* strains isolated in this study. (a): A. baumannii, (b): P. aeruginosa, (c): S. maltophilia, (d): Pseudomonas spp; P. guariconensis; P. entomophila. TIC: Ticarcillin, TIM: Ticarcillin + Clavulanic acid, TZP: Piperacillin + tazobactam, CAZ: Ceftazidime, FEP: cefoxitin, MEM: meropenem, IPM: Imipenem, FF: Fosfomycin, RA: Rifampicin, SXT: Trimethoprim/sulfamethoxazole, AK: Amikacin, CIP: Ciprofloxacin, DO: Doxycyclin, CT: colistin, CN: Gentamicin.

The less spread strains (K. oxytoca [*n* = 5], E. coli [*n* = 8], and *R. ornithinolytica* [*n* = 3]) were mainly resistant to quinolones, aminoglycosides, sulfamides, and fosfomycin. The imipenem ETEST showed that these strains were resistant, with MICs ranging from 1.5 mg/L to >32 mg/L, and they all remained resistant to ertapenem, with MICs ranging from 0.5 mg/L to >32 mg/L. In addition, the nonfermenting GNB isolates showed high resistance to beta-lactams, particularly A. baumannii. These strains also exhibited resistance to other antibiotic families, such as quinolone, sulfamides, and aminoglycosides, with 100% resistance to each. Colistin and fosfomycin were the most effective antibiotics. Moreover, all strains were resistant to carbapenem drugs, namely, imipenem and meropenem, and E-TEST showed that all strains were highly resistant, with MICs of >32 mg/L for both drugs. Furthermore, all strains of P. aeruginosa were sensitive to imipenem and meropenem, and only two strains of *P. guariconensis* were extremely resistant. The phenotypic test results are mentioned in Fig. 3.

### Molecular analyses.

**(i) Molecular mechanisms of carbapenem resistance in *Enterobacterales.*** Of the 76 ertapenem-resistant *Enterobacterales*, 65 strains harbored at least one of the tested carbapenemase encoding genes. The NDM metalloenzyme was the most prevalent carbapenemase, and it was identified in 56 strains, mainly in K. pneumoniae (*n* = 29), which was followed by C. freundii (*n* = 21), *R. ornithinolytica* (*n* = 3), *C. youngae* (*n* = 2), and K. oxytoca (*n* = 1). OXA-48 was detected in 15 strains, mostly in K. pneumoniae (*n* = 11), K. oxytoca (*n* = 3), and C. freundii (*n* = 1). 4 OXA-48 positive strains remained susceptible to imipenem (MICs of ≤2 mg/L). The VIM enzyme was detected in 7 strains. None of the strains harbored the IMP and KPC genes. On the other hand, 11 strains harbored at least two carbapenemase enzymes with three combinations, as follows: *bla*_NDM_ + *bla*_OXA-48_, *bla*_NDM_+ _VIM_, and *bla*_NDM_ + *bla*_OXA-48_+*bla*_VIM_. By sequencing the NDM positive strains, three different variants were identified, namely, NDM-1, NDM-2, and NDM-5. On the other hand, from the VIM positive strains, two different variants were identified, namely, VIM-1 and VIM-4 (Table 1).

**TABLE 1:** Phenotypic and genotypic features of Enterobacteriaceae strains isolated from COVID-19 patients admitted to some isolation centers in the eastern part of Libya^*a*^

Strain code	Strain ID	Antibiotic susceptibility profile	E-TEST ETP (μg/mL)	E-TEST IMP (μg/mL)	Colistin MIC (μg/mL)	Carbapenemase-encoding genes
BMC O44	K. pneumoniae	AX, AMC, FEP, TPZ, CRO, ETP, IMP, FF, F, SXT, AK, CIP, CN, CT	4	3	32	NDM-1
BMC R28(2)		AX, AMC, FEP, TPZ, CRO, ETP, IMP, FF, F, CIP, CN, CT	>32	6	16	NDM-1, OXA-48
BMC R29(2)		AX, AMC, FEP, TPZ, CRO, ETP, IMP, FF, F, CIP, CT	>32	>32	16	NDM-5
NMH R24(2)		AX, AMC, FEP, TPZ, CRO, ETP, IMP, FF, F, SXT, AK, CIP, DO, CT	>32	16	0.5	NDM-5, OXA-48
NMH-N25(3)		AX, AMC, FEP, TPZ, CRO, ETP, IMP, FF, F, SXT, AK, CIP, DO, CN	>32	>16	0.5	OXA-48
NMH-R23		AX, AMC, FEP, TPZ, CRO, ETP, IMP, FF, F, SXT, AK, CIP, DO, CN	>32	16	0.5	NDM-5, OXA-48
MNS-R3		AX, AMC, FEP, TPZ, CRO, ETP, IMP, FF, F, SXT, AK, CIP, DO, CN	>32	>32	0.5	NDM-5
HH-R3(2)		AX, AMC, FEP, TPZ, CRO, ETP, IMP, FF, F, SXT, AK, CIP, DO, CN	>32	>32	1	NDM-5
BMC-R27		AX, AMC, FEP, TPZ, CRO, ETP, IMP, FF, F, SXT, AK, CIP, DO, CN	>32	>32	0.5	NDM-5
NNH-R25		AX, AMC, FEP, TPZ, CRO, ETP, IMP, FF, F, SXT, AK, CIP, DO, CN	>32	16	0.25	NDM-5, OXA-48
NMH-O17(2)		AX, AMC, FEP, TPZ, CRO, ETP, IMP, FF, F, SXT, AK, CIP, DO	>32	8	0.5	OXA-48
MNS-N2		AX, AMC, FEP, TPZ, CRO, ETP, IMP, FF, F, SXT, AK, CIP, CN	>32	>32	0.5	NDM-5
HH-N5 DOUBLE		AX, AMC, FEP, TPZ, CRO, ETP, IMP, FF, F, SXT, AK, CIP, CN	>32	24	0.5	NDM-5, OXA-48
NMH-O23		AX, AMC, FEP, TPZ, CRO, ETP, IMP, FF, F, SXT, AK, CIP, CN	>32	16	0.5	NDM-5, OXA-48
MNS-N6		AX, AMC, FEP, TPZ, KF, CRO, ETP, IMP, FF, F, SXT, AK, CIP	>32	>32	0.5	NDM-5, OXA-48
NMH-N17(3)		AX, AMC, FEP, TPZ, KF, CRO, ETP, IMP, FF, F, SXT, AK, CIP, CN	>32	24	0.5	NDM-5, OXA-48
NMH-R17(2)		AX, AMC, FEP, TPZ, KF, CRO, ETP, IMP, FF, F, SXT, AK, CIP	>32	16	0.5	OXA-48
HH-R8 NMH R8(1)		AX, AMC, FEP, TPZ, KF, CRO, ETP, IMP, FF, F, SXT, AK, CIP	>32	>32	0.5	NDM-5
NMH-R19(2)		AX, AMC, FEP, TPZ, KF, CRO, ETP, IMP, FF, F, SXT, AK, CIP	>32	>32	0.5	NDM-1
NMH-R20		AX, AMC, FEP, TPZ, KF, CRO, ETP, IMP, FF, F, SXT, AK, CIP	>32	16	1	NDM-5
NMH-R18(2)		AX, AMC, FEP, TPZ, KF, CRO, ETP, IMP, FF, F, SXT, AK, CIP	32	>32	0.5	NDM-5
NMH-R26(1)		AX, AMC, FEP, TPZ, KF, CRO, ETP, IMP, FF, F, SXT, AK, CIP	>32	>32	0.5	NDM-1
HH-R7(1)		AX, AMC, FEP, TPZ, KF, CRO, ETP, IMP, FF, F, SXT, AK, CIP	>32	>32	0.5	NDM-1
HH-R4(2)		AX, AMC, FEP, TPZ, KF, CRO, ETP, IMP, FF, F, SXT, AK, CIP	>32	>32	1	NDM-5
HH-N2 NASAL		AX, AMC, FEP, TPZ, KF, CRO, ETP, IMP, FF, F, SXT, AK, CIP	4	6	0.5	NDM-1
HH-R2		AX, AMC, FEP, TPZ, KF, CRO, ETP, IMP, FF, F, SXT, AK, CIP	>32	>32	1	NDM
BMC N44(3)		AX, AMC, FEP, TPZ, KF, CRO, ETP, IMP, FF, F, AK, CIP, DO	4	1.5	0.5	NDM-1
BMC N 50		AX, AMC, FEP, TPZ, KF, CRO, ETP, IMP, FF, SXT, AK, CIP, DO	12	8	0.5	NDM-1
BMC O57(2)		AX, AMC, FEP, TPZ, KF, CRO, ETP, IMP, SXT, CIP, CN	2	3	0.5	NDM-5
BMC N 59(2)		AX, AMC, FEP, TPZ, KF, CRO, ETP, IMP(I), FF, F SXT, AK, CIP, CN	6	2	0.5	NDM-1
BMC-R45(1)		AX, AMC, FEP, TPZ, KF, CRO, ETP, IMP(I), FF, SXT, CIP, CN	6	2	0.5	NDM-1
BMC-O52(2)		AX, AMC, FEP, TPZ, KF, CRO, ETP, IMP(I), FF, SXT, AK, CIP, CN	1.5	1		NDM-1
BMC-N44(2)	C. freundii	AX, AMC, FEP, TPZ, KF, CRO, ETP, IMP(I), FF, F, SXT, AK, CIP, CN	2	2	0.5	NDM-5
BMC-O59(1)		AX, AMC, FEP, TPZ, KF, CRO, ETP, IMP, SXT, CIP	1.5	3	0.5	NDM-1
BMC-N 58(4)		AX, AMC, FEP, TPZ, KF, CRO, ETP, IMP, SXT, CIP, CN	>32	1	0.5	NDM-1
BMC-N 50(4)		AX, AMC, FEP, TPZ, KF, CRO, ETP, IMP, SXT, CIP, DO, CN	1	2	0.5	NDM-1
BMC-O57(2)		AX, AMC, FEP, TPZ, KF, CRO, ETP, IMP SXT, AK, CN	4	12	0.5	NMD-5, VIM-4
NMH-O20(2)		AX, AMC, FEP, TPZ, KF, CRO, ETP, IMP, CIP		8	0.5	NDM-1
HH-R4(1)		AX, AMC, FEP, TPZ, KF, CRO, ETP, IMP, CIP	1	4	0.5	NDM-1
BMC-N46(1)		AX, AMC, FEP, TPZ, KF, CRO, ETP, IMP, AK, CIP	4	6	0.5	NDM-1
BMC N 59(1)		AX, AMC, FEP, TPZ, KF, CRO, ETP, IMP, CIP, CN	2	4	0.5	NDM-2
BMC-O54(1)		AX, AMC, FEP, TPZ, KF, CRO, ETP, IMP, AK	2	2	0.5	NDM-1, VIM-1
BMC-O58(1)		AX, AMC, FEP, TPZ, KF, CRO, ETP, IMP, AK	1.5	3	0.5	NDM-1, OXA-48, VIM-1
BMC-O43(1)		AX, AMC, FEP, TPZ, KF, CRO, ETP, IMP, CIP	>32	>32	0.5	NDM-1
BMC-N55(2)		AX, AMC, FEP, TPZ, KF, CRO, ETP, IMP(I), CIP, CN	2	6	0.5	NDM-1
BMC-N56		AX, AMC, FEP, TPZ, KF, CRO, ETP, IMP(I), SXRT, AK, CIP, DO, CN	16	2	0.5	NDM-1
BMC-O53(2)		AX, AMC, FEP, TPZ, KF, CRO, ETP, IMP(I),	12	3	0.5	VIM-1
BMC-O55(1)		AX, AMC, FEP, TPZ, KF, CRO, ETP, IMP(I), AK, CIP, DO, CN	3	1.5	0.5	NDM-1
BMC-O52(1)		AX, AMC, FEP, TPZ, KF, CRO, ETP, IMP(I), AK, CN	1.5	1	0.5	NDM-1
BMC-O45(2)		AX, AMC, FEP, TPZ, KF, CRO, ETP, IMP(I), SXT, AK, CIP, CN	1	1.5	0.5	NDM-1
BMC-O46		AX, AMC, FEP, TPZ, KF, CRO, ETP, IMP(I),	4	1	0.5	VIM-1
BMC-R 45(2)		AX, AMC, FEP, TPZ, KF, CRO, ETP, IMP(I), CIP	2	6	0.5	NDM-1
NMH-O47(2)		AX, AMC, FEP, TPZ, KF, CRO, ETP, IMP(I), SXT, AK	1	2	0.5	NDM-5
BMC-N52(1)	*C. youngae*	AX, AMC, FEP, TPZ, KF, CRO, ETP, IMP(I), SXT, CIP, CN	1.5	1.5	0.5	NDM-1
BMC-N56(2)		AX, AMC, FEP, TPZ, KF, CRO, ETP, IMP(I), CIP, CN	2	1	0.5	NDM-1
BMC-O53(2)	*C. braakii*	AX, AMC, FEP, TPZ, KF, CRO, ETP, IMP(I), FF, AK, CIP, CN	12	4	0.5	VIM-1
BMC-N53(2)		AX, AMC, FEP, TPZ, KF, ETP, CIP	0.38	0.38	0.5	-^*b*^
BMC O55	E. coli	AX, AMC, FEP, AK, CIP, CT	0.003	0.19	4	S
NMH-N19		AX, AMC, TPZ (I), KF, CRO, SXT, CIP, CT	0.003	0.25	4	S
BMC-N56		AX, AMC, SXT, CIP, CT	0.002	0.25	4	S
BMC-O45		AX, AMC, SXT, CIP, CT	0.002	0.25	4	S
HH-R17		AX, AMC, KF, CRO, FF, F, AK, CT	0.32	0.19	8	S
MNS-N2(3)		AX, AMC, FEP, TPZ, KF, CRO, ETP, IMP, CIP, DO	>32	4	0.5	NDM-5
MNS-R2(3)		AX, AMC, FEP, TPZ, KF, CRO, ETP, IMP, CIP	32	4	0.5	NDM-1
BMC-N52(4)	K. oxytoca	AX, AMC, FEP, TPZ, KF, CRO, ETP, IMP, FF, F, SXT, AK, CIP, DO, CN	8	4	0.5	VIM-1
BMC-N56(4)		AX, AMC, FEP, TPZ, KF, CRO, ETP, IMP, FF, F, SXT, AK, CIP, DO	0.5	0.5	0.5	OXA-48
BMC-N58(1)		AX, AMC, FEP, TPZ, KF, CRO, ETP, IMP, FF, SXT, CIP, CN	1.5	4	0.5	NDM-5
BMC-O43(2)		AX, AMC, FEP, TPZ, KF, CRO, ETP, CIP, CN	0.38	0.38	0.5	OXA-48
BMC-N31(3)		AX, AMC, FEP(I), TPZ, KF, CRO, ETP, FF, SXT	16	0.38	0.5	OXA-48
BMC-N51(2)	*R. ornithinolytica*	AX, AMC, FEP, TPZ, KF, CRO, ETP, IMP, SXT, AK, CIP, DO, CN	>32	12	0.5	NDM-2
BMC-N58(2)		AX, AMC, FEP, TPZ, KF, CRO, ETP, IMP, FF, AK, CIP, CN	6	2	0.5	NDM-1
BMC-O57(2)		AX, AMC, FEP, TPZ, KF, CRO, ETP, IMP, FF, SXT, CIP, CN	1.5	4	0.25	NDM-1
BMC O47	E. cloacae	AX, AMC, FEP, TPZ, KF, CRO, ETP, IMP(I), CIP	4	>32	64	-
BMC O55		AX, AMC, TPZ, KF, CRO, ETP, IMP(I), FF, F, SXT, CIP, CN	>32	1.5	0.5	-
BMC O59		AX, AMC, TPZ, KF, CRO, ETP, FF, F, SXT	1.5	0.75	0.5	-
BMC O46		AX, AMC, TPZ, KF, CRO, ETP, FF, CIP	1	0.5	0.5	-

a O, oral; N, nasal; R, rectal; BMC, Benghazi Medical Center; HH, Hawari Hospital; MNH, New Marwa Hospital; MNS, Mansoura Isolation Center. AX, amoxicillin; AMC, amoxicillin/clavulanic acid; FEP, cefoxitin; TZP, piperacillin-tazobactam; KF, cefalotin; CRO, ceftriaxon; ETP, ertapenem; IMP, imipenem; FF, fosfomycin; F, nitrofurantoin; SXT, trimethoprim/sulfamethoxazole; AK, amikacin; CIP, ciprofloxacin; DO, doxycycline; CN, gentamicin; CT, colistin; WT, wild-type.

b ND, Not Determined.

**(ii) Molecular mechanisms of carbapenem resistance in *non-Enterobacterales.*** Among nonfermenting bacteria, A. baumannii was the most prevalent carbapenem-resistant strain. The OXA-23 enzyme was the most common carbapenemase gene that was detected in 19 isolates, and it was followed by the NDM gene in 9 strains. On the other hand, 8 strains harbored 2 types of carbapenemases, with a combination of *bla*_OXA-23_+*bla*_NDM-1_. Moreover, one strain harbored a VIM-1 enzyme. For the Pseudomonas spp., all P. aeruginosa were sensitive to carbapenem antibiotics, and only two strains of *P. guariconensis* harbored the VIM-2 gene (Table 2).

**TABLE 2:** Phenotypic and genotypic features of non-*Enterobacteriaceae* strains isolated from COVID-19 admitted patients in a center of isolation in Libya^*a*^

Strain code	Strain ID	Antibiotic susceptibility profile	E-TEST MEM (μg/mL)	E-TEST IMP (μg/mL)	Carbapenemase-encoding genes
NMH R 24	*baumannii*	TIC, TIM, TPZ, ATM, CAZ, FEP, MEM, IMP, FF, RA, SXT, AK, CIP, DO, CN	>32	>32	NDM-1, OXA-23
MNH R25 double		TIC, TIM, TPZ, ATM, CAZ, FEP, MEM, IMP, FF, RA, SXT, AK, CIP, DO, CN	>32	>32	OXA-23
BMC N54(1)		TIC, TIM, TPZ, ATM, CAZ, FEP, MEM, IMP, FF, SXT, CIP, DO, CN	>32	>32	VIM-1
BMC R 27(2)		TIC, TIM, TPZ, ATM, CAZ, FEP, MEM, IMP, RA, SXT, AK, CIP, DO, CN	>32	>32	NDM-1
BMC R32(2)		TIC, TIM, TPZ, ATM, CAZ, FEP, MEM, IMP, RA, SXT, AK, CIP, DO, CN	>32	>32	NDM-1, OXA-23
BMC R28(1)		TIC, TIM, TPZ, ATM, CAZ, FEP, MEM, IMP, RA, SXT, AK, CIP, DO, CN	>32	>32	OXA-23
MNS R3(3)		TIC, TIM, TPZ, ATM, CAZ, FEP, MEM, IMP, RA, SXT, AK, CIP, DO, CN	>32	>32	NDM-1, OXA-23
HH-N3(3)		TIC, TIM, TPZ, ATM, CAZ, FEP, MEM, IMP, RA, SXT, AK, CIP, DO, CN	>32	>32	OXA-23
BMC N28		TIC, TIM, TPZ, ATM, CAZ, FEP, MEM, IMP, RA, SXT, AK, CIP, DO, CN	>32	>32	NDM-1, OXA23
BMC N50(1)		TIC, TIM, TPZ, ATM, CAZ, FEP, MEM, IMP, RA, SXT, AK, CIP, DO, CN	>32	>32	NDM-1, OXA-23
NMH O24(2)		TIC, TIM, TPZ, ATM, CAZ, FEP, MEM, IMP, SXT, AK, CIP, DO, CN	>32	>32	OXA-23
NMH N25		TIC, TIM, TPZ, ATM, CAZ, FEP, MEM, IMP, SXT, AK, CIP, DO, CN	>32	>32	OXA-23
NMH O19(1)		TIC, TIM, TPZ, ATM, CAZ, FEP, MEM, IMP, SXT, AK, CIP, DO, CN	>32	>32	OXA-23
NMH N24(2)		TIC, TIM, TPZ, ATM, CAZ, FEP, MEM, IMP, SXT, AK, CIP, DO, CN	>32	>32	NDM-1, OXA-23
NMH O20(2)		TIC, TIM, TPZ, ATM, CAZ, FEP, MEM, IMP, SXT, AK, CIP, DO, CN	>32	>32	OXA-23
BMC O51(2)		TIC, TIM, TPZ, ATM, CAZ, FEP, MEM, IMP, SXT, AK, CIP, DO, CN	>32	>32	OXA-23
NMH O23(2)		TIC, TIM, TPZ, ATM, CAZ, FEP, MEM, IMP, SXT, AK, CIP, DO, CN	>32	>32	OXA-23
BMC O50(1)		TIC, TIM, TPZ, ATM, CAZ, FEP, MEM, IMP, SXT, AK, CIP, DO, CN	>32	>32	OXA-23
BMC O 52(1)		TIC, TIM, TPZ, ATM, CAZ, FEP, MEM, IMP, RA, SXT, AK, CIP, DO, CN	>32	>32	NDM-1, OXA-23
BMC O45(2)		TIC, TIM, TPZ, ATM, CAZ, FEP, MEM, IMP, SXT, AK, CIP, DO, CN	>32	>32	NDM-1, OXA-23
BMC O26(1)		TIC, TIM, TPZ, ATM, CAZ, FEP, MEM, IMP, SXT, AK, CIP, DO, CN	>32	>32	OXA-23
BMC N55(3)	P. aeruginosa	TIC, TIM, TPZ, ATM, CAZ, FEP, RA, SXT, AK, CIP, DO, CN	0.38	2	S
NMH-N 17(2)		TIC, TIM, TPZ, ATM, CAZ, FEP, MEM(I), RA, SXT, AK, CIP, DO, CN	O,75	1.5	S
BMC R 29(1)		TIC, TIM, TPZ, ATM, CAZ, RA, SXT, AK, DO	0.125	2	S
HH-R1(1)		TIC, CAZ, RA, SXT, DO	0.125	2	S
MNS R1(1)		TIC, RA, SXT, DO	0.125	2	S
MNS R7		TIC, RA, SXT, DO	0.125	1	S
HH R3(1)		TIC, TPZ, RA, SXT, DO	0.125	1	S
BMC N45(2)		RA, SXT, DO	0.125	1	S
BMC N54		RA, SXT, DO	0.125	1	S
NMH R17		FEP, RA, SXT, DO	0.125	1	S
HH-R7(2)		RA, SXT, DO	0.125	1	S
HH R4(1)		SXT, DO	0.125	1	S
HH-R6(1)		CAZ, RA, SXT, DO	0.125	1	S
HH-N8		S	0.125	0.75	S
BMC N 54	*P. guaroconensis*	TIC, TIM, TPZ, ATM, CAZ, FEP, MEM, IMP, SXT, CIP, DO, CN	>32	>32	VIM-2
BMC N 59		TIC, TIM, TPZ, ATM, CAZ, FEP, MEM, IMP, SXT, CIP, DO, CN	>32	>32	VIM-2
BMC N 51	*P. entomophila*	TIC, TIM, MEM, FEP, SXT	0.75	1.5	-^*b*^
HH-O6(1)	S. maltophilia	TIC, TIM, TPZ, ATM, CAZ, FEP, MEM, IMP, CN, CT	>32	>32	-
NMH O21(1)		TIC, TIM, TPZ, ATM, CAZ, FEP, MEM, IMP, SXT, AK, CIP, DO, CT	>32	>32	-
BMC O51(1)		TIC, TPZ, ATM, CAZ, FEP, MEM, IMP, RA, SXT, CIP, CT	>32	>32	-
BMC R29(3)		TIM, TPZ, ATM, CAZ, FEP, MEM, IMP, SXT, CIP, DO, CT	>32	>32	-
NMH-R26		TIC, TPZ, ATM, CAZ, FEP, MEM, IMP, SXT, CIP, DO	>32	>32	-

a O, oral; N, nasal; R, rectal; BMC, Benghazi Medical Center; HH, Hawari Hospital; MNH, New Marwa Hospital; MNS, Mansoura Isolation Center. AX, amoxicillin; AMC, amoxicillin/clavulanic acid; FEP, cefoxitin; TZP, piperacillin-tazobactam; KF, cefalotin; CRO, ceftriaxon; ETP, ertapenem; IMP, imipenem; FF, fosfomycin; F, nitrofurantoin; SXT, trimethoprim/sulfamethoxazole; AK, amikacin; CIP, ciprofloxacin; DO, doxycycline; CN, gentamicin; CT, colistin; WT, wild-type.

b ND, Not Determined.

### Molecular mechanisms of colistin resistance.

PCR screening for plasmid-mediated colistin resistance genes (mcr-1, mcr-2, mcr-3, mcr-4, mcr-5, and mcr-8) was performed on all of the strains. The results showed that four strains of E. coli harbored the mcr-1 encoding gene. For these strains, the MICs were 4 mg/L, and they were sensitive to carbapenem antibiotics (imipenem and ertapenem) and also remained resistant, mainly to quinolones and sulfamides (Table 3).

**TABLE 3:** Colistin resistance features among *Enterobacteriaceae* isolated from Libyan COVID-19 patients admitted to some isolation centers in the eastern part of Libya from May to June of 2021^*a*^

Strain code	Strain ID	Antibiotic susceptibility profile	E-TEST ETP (μg/mL)	E-TEST IMP (μg/mL)	Colistin MIC (μg/mL)	Carbapenemase-encoding gene	Colistin resistance gene
BMC O44	K. pneumoniae	AX, AMC, FEP, TPZ, CRO, ETP, IMP, FF, F, SXT, AK, CIP, CN, CT	4	3	32	NDM-1	mgrB (mutations)
BMC R28(2)		AX, AMC, FEP, TPZ, CRO, ETP, IMP, FF, F, CIP, CN, CT	>32	6	16	NDM-1, OXA-48	mgrB (mutations)
BMC R29(2)		AX, AMC, FEP, TPZ, CRO, ETP, IMP, FF, F, CIP, CT	>32	>32	16	NDM-5	mgrB (truncated)
NMH R24(2)		AX, AMC, FEP, TPZ, CRO, ETP, IMP, FF, F, SXT, AK, CIP, DO, CT	>32	16	0.5	NDM-5, OXA-48	mgrB (mutations)
NMH-N25(3)		AX, AMC, FEP, TPZ, CRO, ETP, IMP, FF, F, SXT, AK, CIP, DO, CN	>32	>16	0.5	OXA-48	WT
NMH-R23		AX, AMC, FEP, TPZ, CRO, ETP, IMP, FF, F, SXT, AK, CIP, DO, CN	>32	16	0.5	NDM-5, OXA-48	WT
MNS-R3		AX, AMC, FEP, TPZ, CRO, ETP, IMP, FF, F, SXT, AK, CIP, DO, CN	>32	>32	0.5	NDM-5	WT
HH-R3(2)		AX, AMC, FEP, TPZ, CRO, ETP, IMP, FF, F, SXT, AK, CIP, DO, CN	>32	>32	1	NDM-5	Stop codon
BMC-R27		AX, AMC, FEP, TPZ, CRO, ETP, IMP, FF, F, SXT, AK, CIP, DO, CN	>32	>32	0.5	NDM-5	Stop codon
NNH-R25		AX, AMC, FEP, TPZ, CRO, ETP, IMP, FF, F, SXT, AK, CIP, DO, CN	>32	16	0.25	NDM-5, OXA-48	Stop codon
NMH-O17(2)		AX, AMC, FEP, TPZ, CRO, ETP, IMP, FF, F, SXT, AK, CIP, DO	>32	8	0.5	OXA-48	Stop codon
MNS-N2		AX, AMC, FEP, TPZ, CRO, ETP, IMP, FF, F, SXT, AK, CIP, CN	>32	>32	0.5	NDM-5	Stop codon
HH-N5 DOUBLE		AX, AMC, FEP, TPZ, CRO, ETP, IMP, FF, F, SXT, AK, CIP, CN	>32	24	0.5	NDM-5, OXA-48	WT
NMH-O23		AX, AMC, FEP, TPZ, CRO, ETP, IMP, FF, F, SXT, AK, CIP, CN	>32	16	0.5	NDM-5, OXA-48	WT
MNS-N6		AX, AMC, FEP, TPZ, KF, CRO, ETP, IMP, FF, F, SXT, AK, CIP	>32	>32	0.5	NDM-5, OXA-48	WT
NMH-N17(3)		AX, AMC, FEP, TPZ, KF, CRO, ETP, IMP, FF, F, SXT, AK, CIP, CN	>32	24	0.5	NDM-5, OXA-48	WT
NMH-R17(2)		AX, AMC, FEP, TPZ, KF, CRO, ETP, IMP, FF, F, SXT, AK, CIP	>32	16	0.5	OXA-48	WT
HH-R8 NMH R8(1)		AX, AMC, FEP, TPZ, KF, CRO, ETP, IMP, FF, F, SXT, AK, CIP	>32	>32	0.5	NDM-5	WT
NMH-R19(2)		AX, AMC, FEP, TPZ, KF, CRO, ETP, IMP, FF, F, SXT, AK, CIP	>32	>32	0.5	NDM-1	WT
NMH-R20		AX, AMC, FEP, TPZ, KF, CRO, ETP, IMP, FF, F, SXT, AK, CIP	>32	16	1	NDM-5	WT
NMH-R18(2)		AX, AMC, FEP, TPZ, KF, CRO, ETP, IMP, FF, F, SXT, AK, CIP	32	>32	0.5	NDM-5	WT
NMH-R26(1)		AX, AMC, FEP, TPZ, KF, CRO, ETP, IMP, FF, F, SXT, AK, CIP	>32	>32	0.5	NDM-1	WT
HH-R7(1)		AX, AMC, FEP, TPZ, KF, CRO, ETP, IMP, FF, F, SXT, AK, CIP	>32	>32	0.5	NDM-1	WT
HH-R4(2)		AX, AMC, FEP, TPZ, KF, CRO, ETP, IMP, FF, F, SXT, AK, CIP	>32	>32	1	NDM-5	WT
HH-N2 NASAL		AX, AMC, FEP, TPZ, KF, CRO, ETP, IMP, FF, F, SXT, AK, CIP	4	6	0.5	NDM-1	WT
HH-R2		AX, AMC, FEP, TPZ, KF, CRO, ETP, IMP, FF, F, SXT, AK, CIP	>32	>32	1	NDM	WT
BMC N44(3)		AX, AMC, FEP, TPZ, KF, CRO, ETP, IMP, FF, F, AK, CIP, DO	4	1.5	0.5	NDM-1	WT
BMC N 50		AX, AMC, FEP, TPZ, KF, CRO, ETP, IMP, FF, SXT, AK, CIP, DO	12	8	0.5	NDM-1	Stop codon
BMC O57(2)		AX, AMC, FEP, TPZ, KF, CRO, ETP, IMP, SXT, CIP, CN	2	3	0.5	NDM-5	WT
BMC N 59(2)		AX, AMC, FEP, TPZ, KF, CRO, ETP, IMP, FF, F SXT, AK, CIP, CN	6	2	0.5	NDM-1	Stop codon
BMC-R45(1)		AX, AMC, FEP, TPZ, KF, CRO, ETP, IMP, FF, SXT, CIP, CN	6	2	0.5	NDM-1	Stop codon
BMC-O52(2)		AX, AMC, FEP, TPZ, KF, CRO, ETP, IMP, FF, SXT, AK, CIP, CN	1.5	1		NDM-1	Stop codon
BMC O55	E. coli	AX, AMC, FEP, AK, CIP, CT	0.003	0.19	4	S	Mcr-1
NMH-N19		AX, AMC, TPZ (I), KF, CRO, SXT, CIP, CT	0.003	0.25	4	S	Mcr-1
BMC-N56		AX, AMC, SXT, CIP, CT	0.002	0.25	4	S	Mcr-1
BMC-O45		AX, AMC, SXT, CIP, CT	0.002	0.25	4	S	Mcr-1
HH-R17		AX, AMC, KF, CRO, FF, F, AK, CT	0.32	0.19	8	S	-^*b*^

a O, oral; N, nasal; R, rectal; BMC, Benghazi Medical Center; HH, Hawari Hospital; MNH, New Marwa Hospital; MNS, Mansoura Isolation Center. AX, amoxicillin; AMC, amoxicillin/clavulanic acid; FEP, cefoxitin; TZP, piperacillin-tazobactam; KF, cefalotin; CRO, ceftriaxon; ETP, ertapenem; IMP, imipenem; FF, fosfomycin; F, nitrofurantoin; SXT, trimethoprim/sulfamethoxazole; AK, amikacin; CIP, ciprofloxacin; DO, doxycycline; CN, gentamicin; CT, colistin; WT, wild-type.

b ND, Not Determined.

### Colistin resistance, mediated by alterations of the *mgrB* gene.

The entire *mgrB* gene of the 32 K. pneumoniae strains was amplified via standard PCR and sequenced. Among these 32 K. pneumoniae strains, three strains were resistant to colistin, and their MICs ranged from 16 to 32 mg/L. The results of the sequencing confirmed the resistance to colistin via alterations in the *mgrB* encoding gene (deleterious mutations and a truncated gene) (Table 3).

## DISCUSSION

A new virus has been described by the International Committee on Taxonomy of Viruses (ICTV) as severe acute respiratory syndrome coronavirus (SARS-CoV-2). However, the recent increase in carbapenemase-producing bacteria (CPB) can be attributed, in part, to the SARS-CoV-2 pandemic, long hospitalization, inappropriate use of antibiotics (as empirical therapies or as treatments), and noncompliance to infection prevention and control measures. The inappropriate use of antibiotics has contributed to the wide spread of multidrug-resistant bacteria (9, 10), which are essentially resistant to “carbapenem” antibiotics, which are considered to be the last resorts with which to treat Gram-negative bacterial infections.

In addition, broad-spectrum antibiotics were administered in nearly 70% of SARS-CoV-2 related hospital admissions and in 80 to 100% of SARS-CoV-2 related intensive care unit admissions (5). The impact of the SARS-CoV-2 pandemic on the propagation of antibiotic resistance in health care settings has not yet been fully described.

In this context, we describe the spread of carbapenemase-producing, Gram-negative bacteria, mainly due to carbapenem-resistant *Enterobacterales* (CRE) and A. baumannii (CRAB).

A large study performed in Wuhan Union Hospital emphasized that of the bacteria isolated, Gram-negative bacteria represented 85.5% (11). These findings are in concordance with our results; the most frequently isolated bacteria in our study were K. pneumoniae, C. freundii, and A. baumannii, at 26.89% (32/119), 17.64% (21/119), and 17.64% (21/119), respectively. A study published by Li et al. in 2020 in Wuhan, China, found that the most prevalent isolated strains were A. baumannii (35.8%), K. pneumoniae (30.8%), and S. maltophilia (6.3%). Also, Cano-Martín et al. reported in a study that was published in 2021 that K. pneumoniae, K. oxytoca, C. freundii, E. coli, Enterobacter cloacae, A. baumannii, and P. aeruginosa were the Gram-negative bacteria that were responsible for coinfection in SARS-CoV-2 patients (12).

Furthermore, several reports described the increases rate of multidrug-resistant *Enterobacterales*, particularly K. pneumoniae, suggesting the overuse of antimicrobials during the SARS-CoV-2 pandemic (13). The most prevalent enzyme was the metallo-β-lactamase NDM, occurring mainly by the variant NDM-5 and NDM-1 in K. pneumoniae, at 21.5% and 12.65%, respectively. Several findings have referred to the spread of NDM, especially the NDM-5-producing E. coli strains in the ICU dedicated to SARS-CoV-2 patients in France (14). Our results are in line with another report performed on SARS-CoV-2 patients in Italy, which found that 14/41 patients (34%) were infected with carbapenemase-producing K. pneumoniae (15).

Furthermore, this metallo-β-lactmase was found in *Citrobacter* spp., mainly C. freundii. They are recognized as opportunistic pathogens and cause many health care-associated infections, particularly respiratory infections and urinary tract infections. A case report that was conducted by Du et al. and was published in 2013 suggested the occurrence of NDM-1 via C. freundii isolated from a urine culture from a 63-year-old Chinese male who developed urethral stricture and suffered from dysuria (16). In China, another work highlighted the detection of NDM-1 from a 52-year-old Chinese person (17). On the other side of the world, a recent study conducted in Africa mentioned the isolation of NDM-1 producing C. freundii from a rectal swab of a patient (18). It is of major concern to note that all of the patients in the current study suffered from chronic diseases, including diabetes mellitus, hypertension, and cancer.

Furthermore, OXA-48 was widely described in *Enterobacterales*, and several studies have reported the emergence of the OXA-48 enzyme, including in K. pneumoniae, K. oxytoca, E. coli, E. cloacae, and Morganella morganii (19), but little information is available concerning carbapenemase production in C. freundii (20–22).

The emergence of carbapenem-resistant A. baumannii (CRAB) is a serious concern in clinical practice, as it limits treatment options for patients (23). In 2013, the Centers for Disease Control and prevention (CDC) listed the multidrug-resistant Acinetobacter as a “serious threat”. However, in 2019, carbapenem-resistant A. baumannii (CRAB) has been reported as an “urgent threat pathogen”. This organism causes untreatable infections due to its high antimicrobial resistance and its arsenal of virulence agents, which are responsible for its high case mortality rate (24). Several studies highlighted the major role of multidrug resistant (MDR) A. baumannii and its implication in coinfection in SARS-CoV-2 patients who were admitted into an intensive care unit (ICU) (25). The main concern is that CRAB can transmit resistance determinants through mobile genetic elements between strains in health care settings, thereby limiting the availability of antimicrobial therapeutic options (23).

The current study suggested that OXA-23 was linked to carbapenem resistance, and 19 isolates were observed to be carrying this gene. Worldwide, several findings showed that *bla*_OXA-23_ is the most prevalent gene conferring resistance to carbapenem antibiotics in A. baumannii (26, 27).

Furthermore, *bla*_NDM-1_ was shown in nine strains, and similar findings were reported by Ramadan et al., suggesting the presence of resistance genes other than OXA genes, including NDM-1, TEM, and CTX-M in A. baumannii isolated from SARS-CoV-2 patients (28). Our findings are in concordance with another study conducted by Moubareck et al. in 2020, which found that CRAB harbored the gene encoding OXA-23 carbapenemase as well as an additional carbapenemase gene encoding NDM (29).

The global clinical problem is the increase of multidrug-resistant bacteria, particularly carbapenem-resistant bacteria. Colistin was initially used on humans in 1950 to treat Gram-negative bacterial infections. Later, it was gradually abandoned because of its presumed nephrotoxicity, and it was replaced by new antibiotics with similar a spectrum and effectiveness but without the toxicity, such as the second-generation and third-generation cephalosporins (30). In fact, this has contributed to the reintroduction of colistin into clinical therapy to treat infections caused by multidrug-resistant bacteria (31).

This part of the study describes the occurrence of colistin-resistant *Enterobacterales* (K. pneumoniae and E. coli) in SARS-CoV-2 patients. We found that K. pneumoniae strains carried carbapenem-resistant genes, but this is not the case for E. coli that carry only colistin-resistant genes. It is worth noting that many different genetic modifications were observed in our three colistin-resistant K. pneumoniae *mgrB* negative regulator genes, which resulted from deleterious mutations in two strains and a missing part of the *mgrB* in one strain. Colistin-resistant clinical isolates have been previously reported in the Middle East region, including the Arabian Peninsula, Turkey, and Egypt (32), as well as in Africa (Tunisia and Algeria) (33, 34).

Of note, mcr-1 has rarely been reported in K. pneumoniae, compared with E. coli. Here, we describe the occurrence of mcr-1 in E. coli. Initially, the plasmid-mediated colistin gene mcr-1 was documented in 2015 in China (35). Since then, it has dramatically diffused worldwide, and it has become the most reported pathway implicated in colistin resistance in both animals and humans. So far, 10 MCR variants have been defined, accounting for a varied geographic distribution (36). MCR-1 was widely detected in Europe, Asia, and Africa, mainly in animals and in the environment (wastewater and sewage water), with few findings documenting mcr-1 in clinical isolates (37). To the best of our knowledge, this is the first time that the mobile colistin resistant gene (mcr-1) has been detected in a hospital setting in Libya.

This is the first comprehensive study to investigate the carriage of mcr-1 by *Enterobacterales* among individuals with SARS-CoV-2 in clinical settings in Libya. NDM was the major carbapenemase, with NDM-1 and NDM-5 being the most dominant types. The strains that were positive for mcr-1 were all E. coli, and they remained sensitive to carbapenem antibiotics. For K. pneumoniae, resistance to colistin was enhanced by *mgrB* modification.

Increased surveillance, continuous epidemiological monitoring at the local and national scales, the application of hygiene practices, and, above all, the rational use of carbapenem antibiotics and, recently, colistin (according to the dosage) are required to reduce the propagation of MDR strains and to maintain the efficacy of these antibiotics.

## MATERIALS AND METHODS

### Data collection and patients.

This study was performed in SARS-CoV-2 isolation centers in the eastern part of Libya (Benghazi and Shahat), in different hospital settings (Benghazi Medical Center, New Marwa Hospital, Hawari Hospital, and Mansoura Hospital), from May to June of 2021. Patients (>40 years) were admitted to these hospital settings. Moreover, laboratory confirmations of SARS-CoV-2 infections were performed, following the Gene Expert protocol of nasopharyngeal swabs. 109 samples were collected from 43 patients. The swabs were recovered from the oral cavity (*n* = 35), nasal cavity (*n* = 45), and rectal cavity (*n* = 29). Patients’ clinical information underlying diseases, comorbidities, age, and gender are summarized (data not shown).

### Swab collection, culture media, and bacterial isolation.

The sampling was performed using sterile cotton swabs. The swabs were kept on transport media. These swabs were stored at 4°C and were transported to the IHU Mediterranean Infection in order to achieve the isolation steps in the laboratory. The collected samples were incubated in 10 mL of brain heart infusion broth medium (bioMérieux, Marcy l’Etoile, France) for 48 or 72 h at 37°C. The broth samples were then cultured on MacConkey agar medium (bioMérieux, Marcyl’Etoile, France) that was supplemented with cefotaxime (2 mg/L) and ertapenem (2 mg/L) (Bio-Rad, Marnes-la-Coquette, France) in order to isolate third-generation cephalosporins (3GC) and carbapenem-resistant Gram-negative bacteria. To detect colistin resistance, the swabs were cultured on Lucie Bardet-Jean-MarcRolain (LBJMR) medium. This medium is Purple Agar Base (31 g/L) that is supplemented with colistin (4 mg/L), vancomycin (50 mg/L), and glucose (7.5 g/L) as a fermentative substrate (38).

Following 24 h of incubation at 37°C, the cultures yielded morphologically different colonies (differing in color, size, and shape) on each plate. Well-isolated colonies that were growing on the selective media were taken separately and purified.

The identification of pure isolates was performed via the matrix-assisted laser desorption/ionization time-of-flight mass spectrometry (MALDI-TOF-MS) method (Microflex; Bruker Daltonics, Bremen, Germany), with the flex control and the Biotyper 3.0 software (Bruker Daltonics).

### Antibiotic susceptibility testing.

Antibiotic susceptibility testing was carried out on Mueller-Hinton agar (bioMérieux, Marcy l’Etoile, France), using the standard disk diffusion method, according to the Antibiogram Committee of the French Society for Microbiology (CA-SFM/EUCAST 2021) (http://www.sfm-microbiologie.org/). 2 different panels of 16 antibiotic disks (Bio-Rad, Marnes-la-Coquette, France) were used for lactose fermenting and for nonfermenting GNB as follows. For the lactose fermenting GNB, the antibiotics used were amoxicillin (AX), amoxicillin-clavulanic acid (AMC), cefoxitin (FEP), piperacillin + tazobactam (TZP), cephalothin (KF), ceftriaxone (CRO), ertapenem (ETP), imipenem (IPM), colistin (CT), amikacin (AK), gentamicin (CN), ciprofloxacin (CIP), fosfomycin (FF), nitrofurantoin (F. NIT), doxycycline (DO), and trimethoprim-sulfamethoxazole (SXT). For the nonfermenting GNB: ticarcillin (TIC), ticarcillin + clavulanic acid (TIM), piperacillin + tazobactam (TZP), ceftazidime (CAZ), cefoxitin (FEP), imipenem (IPM), meropenem (MEM), colistin (CS), amikacin (AK), gentamicin (CN), ciprofloxacin (CIP), fosfomycin (FF), doxycycline (DO), trimethoprim-sulfamethoxazole (SXT), and rifampicin (RA).

Furthermore, MICs were performed via the E-TEST strip (bioMérieux, Marcy l‘Etoile, France) method for carbapenem-resistant isolate screening. The MIC results were interpreted using the CLSI breakpoints. The results of colistin susceptibility were validated using MIC by UMIC, according to the joint CLSI-EUCAST Polymyxin Breakpoints Working Group.

### Molecular characterization of resistance genes.

**(i) Carbapenem resistance genes.** The molecular characterization of carbapenemase-encoding genes (*bla*_KPC_, *bla*_IMP_, *bla*_VIM_, *bla*_NDM_, *bla*_OXA-48_, *bla*_OXA-23_, *bla*_OXA-24_, and *bla*_OXA-58_) was performed via real-time PCR, and positive results were confirmed via conventional PCR, using specific primers, as described previously (39). The amplicons of each tested gene were sequenced via BigDye1 terminator chemistry on an automated ABI 3130 sequencer (PE Applied Biosystems, Foster City, CA, USA), based on the Sanger method. The sequences were compared with those that are available in the GenBank database. The sequences were then aligned using the npsa-clustal W, the Network Protein Sequence Analysis (https://npsa-prabi.ibcp.fr/), to analyze the amino acid sequences that were translated into proteins. The PROVEAN software package (http://provean.jcvi.org/index.php) was used to check whether amino acid sequence changes could induce alterations of protein functions.

**(ii) Colistin resistance genes.** All of the strains were screened for the presence of plasmid-mediated colistin resistance genes, represented by mcr-1, mcr-2, mcr-3, mcr-4, mcr-5, and mcr-8 via PCR, using specific primers and probes. K. pneumoniae strains were investigated to detect possible genetic alterations associated with colistin resistance in the *mgrB* genes. The *mgrB* genes were sequenced using a BigDye Terminator Cycle Sequencing Kit (Applied Biosystems, Foster City, CA, USA). Sequences of *mgrB* were obtained and compared to that of the reference strain of K. pneumoniae MGH78578, which carries wild-type genes (GenBank accession number: NC_009648). The PROVEAN software package (http://provean.jcvi.org/index.php) was used to check whether amino acid sequence changes could induce alterations of protein functions (40).
